# Could the Risk of Chronic Degenerative Valve Disease (CDVD) in Dogs Depend on Exposure to Endocrine-Disrupting Chemicals Polluting the Environment?

**DOI:** 10.3390/ani15233443

**Published:** 2025-11-28

**Authors:** Krystyna Makowska, Julia Martín, Robert Pasławski, Andrzej Rychlik, Irene Aparicio, Juan Luis Santos, Esteban Alonso, Małgorzata Górecka-Politańska, Sławomir Gonkowski

**Affiliations:** 1 Department of Clinical Diagnostics, Faculty of Veterinary Medicine, University of Warmia and Mazury in Olsztyn, Oczapowskiego 14, 10-957 Olsztyn, Poland; 2 Departamento de Química Analítica, Escuela Politécnica Superior, Universidad de Sevilla, C/Virgen de África, 7, E-41011 Sevilla, Spain; jbueno@us.es (J.M.); jlsantos@us.es (J.L.S.);; 3 Faculty of Veterinary Medicine, University of Agriculture in Krakow, Mickiewicza 21, 31-120 Krakow, Poland; r.paslawski@umk.pl; 4 Companion Animal Veterinary Clinic, Faculty of Veterinary Medicine, University of Warmia and Mazury in Olsztyn, Oczapowskiego 14, 10-957 Olsztyn, Poland; 5 Department of Clinical Physiology, Faculty of Veterinary Medicine, University of Warmia and Mazury in Olsztyn, Oczapowskiego 13, 10-957 Olsztyn, Poland

**Keywords:** bisphenol A, benzophenones, parabens, PFASs, heart, environmental pollution

## Abstract

Endocrine-disrupting chemicals (EDCs) pollute the environment and food, adversely affecting living organisms. It is known that exposure to EDCs increases the risk of various diseases in humans, but in veterinary medicine, the pathogenic impact of these substances is usually marginalised. Therefore, the aim of the present study was to determine whether exposure to EDCs is associated with a higher risk of chronic degenerative valve disease (CDVD) in dogs. The study included hair samples collected from 60 male and female dogs of various breeds and ages ranging from 9 months to 15 years. Hair samples collected from healthy dogs and dogs with CDVD were analysed for the presence of 15 EDCs (including bisphenol A, parabens, benzophenones and polyfluoroalkyl substances) using liquid chromatography and tandem mass spectrometry (LC-MS/MS) technique. Concentration levels of methylparaben, ethylparaben, propylparaben, perfluoroheptanoic acid and perfluorooctanoic acid in samples collected from dogs with CDVD were statistically significantly higher than those noted in healthy dogs. Differences in concentrations of other EDCs studied between ill and healthy dogs were not statistically significant. The results may suggest that greater exposure of dogs to certain EDCs (such as methylparaben, ethylparaben, propylparaben, perfluoroheptanoic acid, and perfluorooctanoic acid) may increase the risk of CDVD, but further comprehensive clinical and toxicological research is necessary to fully clarify this issue.

## Simple Summary

Endocrine-disrupting chemicals (EDCs) are substances commonly used in various branches of industry. EDCs pollute the environment and food and may adversely affect the living organisms. It is known that exposure to EDCs increases the risk of various diseases in humans, but in veterinary medicine, the pathogenic impact of these substances is usually marginalised. However, it is relatively well known that pet animals, which live in the same conditions as humans and are exposed to similar environmental pollutants, may be treated as sentinels of human exposure to these pollutants. Therefore, the aim of the present study was to determine whether exposure to EDCs is associated with a higher risk of chronic degenerative valve disease (CDVD) in dogs. The obtained results may suggest that greater exposure of dogs to certain EDCs (such as methylparaben, ethylparaben, propylparaben, perfluoroheptanoic acid, and perfluorooctanoic acid) may increase the risk of CDVD, but further comprehensive clinical and toxicological research is necessary to fully clarify this issue.

## 1. Introduction

Endocrine-disrupting chemicals (EDCs) used in the industry commonly pollute the environment [1,2]. Among EDCs, bisphenol A (BPA), parabens (PBs), benzophenones (BPs), and per- and polyfluoroalkyl substances (PFASs) are the most important in toxicology [3,4,5,6]. BPA and PFASs are used as plasticisers [4,5], while PBs exhibit antibacterial and antifungal properties and are components of cosmetics and care products [3]. BPs absorb UV rays and are used in products that must be resistant to sunlight [6].

EDCs affect the endocrine, reproductive, neuronal, and immune systems [3,4,5,6] and exhibit proinflammatory, carcinogenic, and genotoxic activities [7,8,9,10]. BPA, PBs, BPs, and PFASs may also affect the heart, causing cardiac remodelling, heart failure, disorders of the cardiac conduction system, and cardiac rhythm disturbances [11,12,13]. However, to date, no studies have examined the correlation between exposure to EDCs and the risk of heart disease in dogs. Dogs living in close proximity to humans are highly exposed to EDCs, which may be present in commercial food, indoor dust, animal cosmetics, and toys [14,15,16,17]. On the other hand, the cardiac problems in dogs are becoming an increasing challenge for modern veterinary medicine [18,19].

The most common form of heart disease in dogs is chronic degenerative valve disease (CDVD) [20,21,22], in which valves become abnormally thick and have a nodular, lumpy appearance [20,22,23,24]. The causes of CDVD are unknown, but they are associated with aging and a genetic component [20,22]. Some studies suggest that the neurohormonal system and oxidative stress reactions are involved in CDVD development [25,26,27]. However, the influence of EDCs on valve degeneration cannot be ruled out, as some EDCs affect valve function and structure [28].

In biomonitoring of EDCs, hair samples are becoming increasingly important [29,30,31,32]. The hair is collected stress-free and painlessly [29,32]. EDC levels in hair most accurately reflect long-term exposure [33], which is the most important factor affecting the cardio-vascular system [34,35].

This study aimed to determine, for the first time, whether the degree of long-term exposure to EDCs (assessed by EDC levels in hair) may be a factor increasing the risk of CDVD in dogs. The research covered 15 EDCs most frequently polluting the environment, i.e., BPA, PBs (methylparaben—MeP, ethylparaben—EtP, buthylparaben—BuP, propylparaben—PrP), BPs (benzophenone 1—BP-1, benzophenone 2—BP-2, benzophenone 3—BP-3, benzophenone 8—BP-8) and PFASs (five perfluoroalkyl carboxylic acids: perfluorobutanoic acid—PFBuA, perfluoropentanoic acid—PFPeA, perfluorohexanoic acid—PFHxA, perfluoroheptanoic acid—PFHpA, perfluorooctanoic acid—PFOA, as well as perfluorooctane sulfonic acid—PFOS). The results may contribute to a better understanding of the causes of CDVD and the roles of EDCs in this disease.

## 2. Materials and Methods

### 2.1. Chemical Reagents

During the present study, the following reagents were used: standards: BPA (≥98%), MeP (≥99.0%), EtP (≥99.0%), PrP (≥99.0%), BuP (≥99.0%), PFBuA (98%), PFPeA (97%), PFHxA (≥97%), PFHpA (99%), PFOA (96%) and PFOS (≥98%), BP-1 (99%), BP-2 (97%), BP-3 (98%) and BP-8 (≥98%)—all from Sigma-Aldrich (Steinheim, Germany); internal standards (ISs): BPA-d14 (99.4%) from Dr. Ehrenstorfer (Augsburg, Germany), perfluorooctanoic acid-13C4 (PFOA-13C4) (99%), ethylparaben-d5 (EtP-d5) and isotopically labelled BP (BP-d10)—all from Cambridge Isotope Laboratories (Tewksbury, MA, USA), analytical grade acetic acid (HAc), ammonium acetate and sodium dodecyl sulphate (SDS)—all from Panreac (Barcelona, Spain); LC-grade acetone, methanol (MeOH) and water—all from Romil (Barcelona, Spain). Individual stock standard solutions (1000 mg/L) were prepared in MeOH and stored at −18 °C. Working solutions were freshly prepared weekly by diluting the stock solution in MeOH and stored at 4 °C.

### 2.2. Hair Sample Collection and Preparation for Analysis

The investigation included hair samples collected from 60 male and female dogs of various breeds and ages ranging from 1.5 to 15 years. The dogs were the patients of various veterinary clinics in Olsztyn (Poland), divided into two groups of 30 animals each: group H (clinically healthy animals—patients coming to the vet for vaccinations, check-ups, and care treatments, no abnormalities in echocardiographic examination) and group C (animals with diagnosed CDVD). A CDVD diagnosis was based on standard veterinary procedures and included at least one of the following symptoms: rapid and shallow breathing at rest, coughing or gagging, fatigability, fainting, ‘sphinx position’ during sleep, apathy and loss of appetite which led to a visit to the veterinarian. Moreover, upon auscultation, the presence of a systolic heart murmur that was best heard on the left side of the chest at the apex of the heart was a symptom of CDVD. Finally, echocardiography was performed to evaluate the size of individual heart chambers, myocardial contractility and the size of the regurgitant wave. Patients with CDVD included in the study showed stage B, B1 or B2 of this disease, according to the American College of Veterinary Internal Medicine (ACVIM) Specialty of Cardiology [36]. The study only included dogs with a newly diagnosed disease that had not been treated previously.

Because sample collection was completely non-invasive and painless and was performed during veterinary treatments, this research did not require the consent of the Ethical Committee, which is in accordance with the Act for the Protection of Animals for Scientific or Educational Purposes of 15 January 2015 (Journal of Laws of 2015, item 266 with further amendments), applicable in the Republic of Poland. Simultaneously, animal owners were informed about sampling and planned investigations, and sample collection depended on their verbal consent to the study.

Hair samples (about 2 g) were collected from January to December 2021 from the same place (abdomen) of each dog included in the study. Hair was cut with metal scissors as close to the skin as possible. Immediately after sampling, hair was wrapped in aluminium foil and stored in the dark at room temperature. Before analysis, external contamination from the hair was removed by four washes in ultrapure deionised water, SDS (0.1%, *w*/*v*), and again twice in ultrapure water. Between particular washes, the samples were sonicated (5 min). The hair was then dried, cut into small fragments (about 2–3 mm) and stored the dark at room temperature until further analysis.

### 2.3. Hair Sample Analysis

The substances studied were extracted and analysed according to methods previously described by Makowska et al. [30,31], Martin et al. [37], and Rodríguez-Gomez et al. [38]. One hundred milligrams of washed hair was transferred into 10 mL screw-cap glass centrifuge tubes, and after adding 12.5 ng of ISs and 2 mL MeOH/HAc (85:15, *v*/*v*), the tubes were incubated at 38 °C overnight. Samples were then cooled to room temperature, supplemented with acetone (3 mL), sonicated (15 min) and centrifuged (2900× *g*, 10 min). Supernatants were separated, transferred to clean tubes and evaporated to dryness under a nitrogen stream. The extracts were then reconstituted with 0.25 mL of MeOH and filtered through a 0.22 μm nylon filter. After this, a 10 μL aliquot of the extract was injected into the liquid chromatography–tandem mass spectrometry instrument (LC-MS/MS) (Agilent, Santa Clara, CA, USA).

Chromatographic separation was performed using a HALO C-18 Rapid Resolution column with 50 × 4.6 mm i.d. and 2.7 μm particle size (Advanced Materials Technology, Wilmington, DE, USA). The mobile phase was composed of a 10 mM ammonium acetate solution (solvent A) and MeOH (solvent B) at a flow rate of 0.6 mL/min. The gradient program was as follows: 0–14 min, linear gradient from 28% to 70% solvent B; from 70% to 80% solvent B in 5 min, and then increased to 100% solvent B in 6 min and held for 2 min. The column temperature was maintained at 30 °C.

The LC system was coupled to a 6410 triple quadrupole mass spectrometer (MS/MS) (Agilent, Santa Clara, CA, USA) with an electrospray ionisation source operated in negative mode. Two multiple reaction monitoring (MRM) transitions were selected for each compound analysed for quantification and confirmation purposes. The mass spectrometer settings and validation parameters are summarised in Table 1 and Table 2.

### 2.4. Quality Assurance and Quality Control

During the study, a quality assurance/quality control protocol was used. It included the use of control spiked samples, solvent injections, standards containing a mixture of the target compounds and procedural blanks (processed in the same way as the samples) into each analytical batch (15 samples). No quantifiable amounts of the analysed compounds were detected in blank samples.

### 2.5. Statistical Analysis

The statistical analysis was carried out using GraphPad Prism version 9.2.0 (GraphPad Software, San Diego, CA, USA) and included descriptive statistics with evaluation minimum, 25% percentile, median, 75% percentile, maximum, mean ± standard deviation (SD), geometric mean and percent of samples > MQL and MDL. Moreover, a nonparametric Mann–Whitney test was used to compare the levels of the compounds studied between groups H and C. The differences were considered statistically significant at *p* < 0.05 and were indicated with * for *p* < 0.05, ** for *p* < 0.01, *** for *p* < 0.001 and **** for *p* ≤ 0.0001. Concentration levels below MQL were included in the statistics as MQL/2, and those below MDL were included as MDL/2.

## 3. Results

EDCs were detected in both hair samples collected from healthy dogs and from dogs with CDVD. At least three of the tested EDCs were detected at levels above the MQL in every sample included in the study. EDC levels clearly depended on the type of substance and on whether the dog was healthy or suffered from CDVD. Moreover, clear differences in levels of some EDCs were observed even within the same group of dogs. The results for individual groups of substances studied are presented below, and a summary is shown in Table 3.

### 3.1. Levels of PBs and BPA

Levels of all PBs analysed were higher than the MDL in all hair sample studies. Among PBs, MeP and EtP concentrations above MQL were detected in all samples, both in healthy dogs and in dogs with CDVD. MeP mean concentration levels were the highest among all EDCs included in this study and amounted to 76.07 ± 81.97 ng/g (median 44.2 ng/g) in healthy dogs and 162.8 ± 156.9 ng/g (median 105.5 ng/g) in dogs with CDVD. Mean levels of EtP were slightly lower and equalled 25.06 ± 41.61 ng/g (median 11.3 ng/g) and 96.39 ± 199.6 ng/g (median 33.65 ng/g) in groups H and C, respectively. Concentration levels of MeP and EtP differed significantly between healthy dogs and dogs with CDVD (Table 3). The third paraben whose levels differed significantly between the two groups of dogs was PrP. Its levels above MQL were observed in 53.3% in group H and 90% in group C. Mean concentrations of PrP were lower compared to MeP and EtP and equalled 15.55 ± 27.13 ng/g (median 5.38 ng/g) in healthy dogs and 95.47 ± 132.5 ng/g (median 58.70 ng/g) in dogs suffering from CDVD. Mean levels of BuP in group H were similar to those noted in the case of PrP and amounted to 16.83 ± 32.36 ng/g (median 5.38 ng/g), but in dogs with CDVD, they were the lowest among all PBs studied and amounted to 42.64 ± 101.5 ng/g (median 13.95 ng/g). Differences in BuP concentration levels between groups H and C were not statistically significant (*p* = 0.0788).

Compared to parabens, BPA levels above the MQL were observed in a lower percentage of samples (43% in group H and 40% in group C). Therefore, medians of BPA in both groups were below MQL. In healthy dogs, the mean BPA level reached 27.41 ± 39.44 ng/g and was lower than that in dogs with CDVD (56.32 ± 122.7 ng/g). However, these differences were not statistically significant (Table 3).

### 3.2. Levels of BPs

BP-3 was the only benzophenone among those included in the study whose levels in all samples exceeded the MDL. In turn, levels above MQL were observed in 96.7% of samples collected from healthy dogs and in 100% of samples from dogs with CDVD. The mean concentration of BP-3 amounted to 33.68 ± 34.63 ng/g (median 21.75 ng/g) and 57.44 ± 54.62 ng/g (median 38.35 ng/g) in dog groups H and C, respectively. However, differences between both groups were not statistically significant (*p* = 0.0616). Levels of other BPs above MDL and MQL were observed in a smaller percentage of samples. BP-1 levels above MDL were observed in 46.6% and above MQL in 36.6% in both groups (the median was below MQL in both groups). Its mean level was 9.81 ± 25.37 ng/g in healthy dogs and was lower than in dogs with CDVD (41.25 ± 76.81 ng/g), but these differences were not statistically significant. BP-8 and BP-2 in levels above MQL were found in a very small percentage of samples. BP-8 at levels above MQL was detected in 10% of samples from healthy dogs and in 6.7% of samples from dogs with CDVD. In turn, BP-2 levels above MQL were not observed in any of the samples included in the study (Table 3).

### 3.3. Levels of PFASs

Among PFASs, only PFPeA was detected at levels above the MQL in all samples included in the study. Simultaneously, PFPeA levels were the highest among the PFASs analysed. Its mean concentration in healthy dogs was 36.76 ± 113.5 ng/g, which was higher than in dogs with CDVD (27.06 ± 36.80 ng/g). In the case of the median, the situation was reversed. The median PFPeA concentration was 10.65 ng/g in group H and 17.8 ng/g in group C (Table 3). Concentration levels of other PFASs studied were much lower. Mean levels of PFOA (which were noted in levels above MQL in 96.7% of samples from group H and 100% of samples from group C) amounted to 3.62 ± 2.12 ng/g (median 3.13 ng/g) in healthy dogs and 4.88 ± 2.49 ng/g (median 4.35) in dogs with CDVD. These differences between groups were statistically significant (*p* = 0.0361). Similarly to PFOA, statistically significant differences between dog groups H and C (*p* = 0.0384) were also observed for PFHpA, with levels above MQL in 30% of samples from healthy dogs and 60% from dogs with CDVD. Mean levels of PFHpA reached 1.58 ± 3.17 ng/g (median < MQL) and 1.71 ± 2.07 ng/g (median 1.02 ng/g) in groups H and C, respectively. In turn, in the case of PFBuA, the situation was similar to that observed in PFPeA. The mean level of PFBuA (which was observed above the MQL in 63.3% of samples in both groups) in group H was 2.39 ± 3.5 ng/g, higher than in group C (1.97 ± 2.7 ng/g). However, the median PFBuA level in group H (1.06 ng/g) was lower than in group C (1.19 ng/g). Differences in PFBuA levels between both groups were not statistically significant. Mean levels of PFHxA amounted to 2.91 ± 4.16 ng/g (median 0.97 ng/g) and 3.20 ± 2.71 ng/g (median 2.75 ng/g) in groups H and C, respectively. In turn, in the case of PFOS, these values were 1.31 ± 1.09 ng/g (median 0.92 ng/g) in healthy dogs and 2.16 ± 2.94 ng/g (median 1.49 ng/g) in dogs with CDVD. Concentration levels of PFHxA and PFOS between dog groups were not statistically significant (Table 3). PFHxA levels above the MQL were found in 50% of samples collected from group H and 70% of samples from group C. In the case of PFOS, these percentages reached 73.3% and 90%, respectively.

### 3.4. The Influence of Physiological Factors Such as Age and Gender on the EDC Level

In both healthy animals and those diagnosed with CDVD, the influence of environmental factors, such as gender and age, on the levels of the compounds studied was analysed. The results are presented in Supplementary File Tables S1–S4. Within the group of healthy animals, 10 dogs were considered young (under 3 years old), 14 were between 3 and 9 years old, and 6 were considered old (over 9 years old). In this group of animals, the results showed statistically significant differences in age and BuP level, with the highest level observed in young animals (32.99 ± 5.35 ng/g) and the lowest in old dogs (1.52 ± 1.57 ng/g). Among the animals with diagnosed CDVD, 3 were under 3 y.o., 11 were between 3 and 9 y.o., and 16 were over 9 y.o. Within this group, statistically significant differences were observed in the levels of PFOA and PFOS, with the highest levels of those compounds observed in animals aged 3–9 years, and the lowest in young animals under 3 years old. Mean levels of PFOA were 2.31 ± 1.00 ng/g, 6.6 ± 2.57 ng/g and 4.18 ± 1.85 ng/g in young, middle-aged, and old animals, respectively. However, the mean level of PFOS in young animals was <MQL; in middle-aged animals, it was 3.33 ± 4.6 ng/g; and in older animals, it was 1.65 ± 0.92 ng/g.

During the analysis of the dependency between gender and the levels of the studied compounds, only statistically significant differences were observed among healthy animals. This group included 15 males and 15 females. Statistically significant differences were observed for BP1 and BP3, with higher mean levels in males than in females. Mean levels of BP1 reached 16.04 ± 34.06 ng/g in males and 3.58 ± 9.51 ng/g in females, and in the case of BP3, those values were up to 47.59 ± 42.92 ng/g and 19.76 ± 15.06 ng/g in males and females, respectively. In the group of animals diagnosed with CDVD (18 males and 12 females), no statistical differences were found between gender and the levels of the compounds studied.

## 4. Discussion

The present research has confirmed that dogs are significantly exposed to various EDCs, and the degree of this exposure clearly depends on the type of substance. It is consistent with previous studies that have described selected EDCs in dog serum, hair, and faeces [17,30,31,32,39]. The relatively high dog exposure to EDCs results from the fact that these animals live near humans in environments heavily polluted by anthropogenic substances. Of course, as previous research has shown, the degree of exposure of humans and companion animals to EDCs clearly depends on the region where observations have been conducted [30,31,37,40,41]. It is mainly connected with urbanisation and industrialisation, which influence EDC levels in the environment, as well as with human lifestyle, dietary habits, the use of cosmetics, the type of furnishings in homes, and others [42,43,44]. The influence of these local factors on the degree of exposure to EDCs is reflected in markedly different EDC levels in individuals (both humans and animals) from the same area [30,31,37], as observed in the present study in dogs from the same groups. Interestingly, comparisons across studies have shown that EDC levels in dogs are sometimes higher than those reported in humans [30,31,41,45]. This suggests that companion animals are exposed to EDCs more than humans, possibly due to their smaller size and greater exposure to indoor dust, which may be contaminated with high levels of EDCs [46]. Moreover, the use of the most harmful EDCs in the production of human toys, cosmetics, or food containers is limited, unlike in products intended for animals. Companion animals are also usually less mobile than humans, and they spend a large part of their lives in one place. Therefore, it is believed that such animals may serve as a sentinel species for assessing human exposure to various indoor contaminants [47,48].

The present study has confirmed that hair may be used to evaluate long-term exposure of dogs to various EDCs. To date, this matrix has been primarily used in human biomonitoring investigations [37,38,40,41,45], and studies of dogs are relatively limited [30,31,32]. It should be underlined that hair is a specific matrix. Unlike urine or blood serum, in which EDC levels change rapidly over a short period (even within a few hours), these substances are deposited in hair and remain there until they fall out [33]. Therefore, hair appears to be the best matrix for evaluating long-term exposure. On the other hand, pollutants may penetrate the hair both through the blood vessels and hair bulb as well as directly from the environment, and therefore, the exact correlation between the levels of substances in the hair and the actual exposure of the body (for example, through the digestive system) poses some difficulties [33]. Nevertheless, both previous studies [30,31,32] and the present research have shown that hair is useful for EDC biomonitoring in dogs.

EDCs, due to their strong, multidirectional adverse effects, are the focus of numerous epidemiological studies in humans, which describe correlations between exposure levels and the risk of various metabolic, hormonal, cardiovascular, and neurological diseases [3,4,5,6]. To date, there have been no such studies in animals, and the role of EDCs polluting the environment as a potential cause of animal diseases is rather marginalised. Knowledge about companion animal exposure to EDCs is limited to the evaluation of levels of these compounds in animal tissues and to correlations between EDC levels and age, gender, lifestyle, diet, and some haematological parameters [30,31,32,49]. Therefore, the present study is the first research on the eventual influence of exposure to EDCs on the risk of the concrete disease entity in companion animals.

It should be noted that the exact reasons for CDVD, which accounts for approximately 75% of all heart disease in dogs, still remain unknown [20,22,24]. It is known that chronic degenerative processes in CDVD are not associated with inflammatory processes but are linked to aging and occur more often in some breeds, such as the Cavalier King Charles spaniel [20,22,24]. Therefore, it is assumed that CDVD may be associated with a familial or inherited genetic component in some breeds, although the specific genetic evidence for the majority of cases is lacking [20,21,22,23,24]. Moreover, probably disturbances in neurochemical regulation of the heart may increase the risk of CDVD [25,50]. It cannot be ruled out that environmental factors (including exposure to EDCs may also contribute to the development of this disease, especially since the influence of EDCs on the heart has been observed in experimental animals [28,51,52], as well as relationships between exposure to EDCs and heart disorders have been demonstrated in humans [53,54]. The present results seem to partially support this hypothesis.

The most visible, statistically significant changes between healthy animals and animals with CDVD observed during the present study concerned methyl-, ethyl-, and propylparaben, suggesting that exposure to these substances may be associated with the risk of CDVD. This aligns with previous studies, including epidemiological observations in humans and experimental investigations that describe the influence of PBs on the heart. Specifically, it is known that human exposure to PBs is significantly correlated with cardiometabolic diseases [55] and alterations in glucose and lipid metabolism that may contribute to cardiac pathological processes [56,57,58]. Although disturbances in glucose metabolism are not specifically cited as a risk factor predisposing to the development of CDVD in dogs, some studies in this species have reported associations between glucose levels and left ventricular diastolic dysfunction [59]. Moreover, experimental investigations have shown that PBs exhibit cardiotoxic effects, including, among others, an influence on cardiomyopathy-related gene transcription [60].

In the present study, statistically significant differences between healthy dogs and dogs with CDVD were also observed for selected PFASs. This fact may confirm, as previously reported, the PFAS-induced influence on cardiac remodelling [61]. Moreover, it is known that chronic exposure to PFASs is connected with an increased risk of cardiovascular events in humans [35]. In turn, experimental studies have shown that PFOA induces histopathological changes in the heart, causes cardiac defects, and dysregulates the expression of genes involved in cardiac development [62,63]. On the other hand, epidemiological studies in humans have not confirmed a strong association between elevated PFAS levels in blood plasma and the risk of cardiovascular disease [11]. Therefore, the roles of PFASs in the development of cardiac diseases remain unclear, as confirmed by the present results, which showed statistically significant differences between healthy dogs and dogs with CDVD only for PFOA and PFHpA. In the case of other PFASs studied, statistically significant differences in their levels between healthy and sick dogs were not observed.

Statistically significant differences in levels of BPA and BP-3 (the only one of the tested BPs studied, whose presence was found in most samples) between healthy animals and animals with CDVD have not been observed, which might seem a bit strange considering the relatively well-known negative effects of these compounds (especially BPA) on the heart. It is known that BPA levels are associated with various types of cardiovascular and cardiometabolic diseases, including hypertension, heart attack and coronary and peripheral arterial disease [54,64]. Moreover, chronic exposure to BPA increases the risk of developing cardiovascular diseases through cardiac remodelling, atherosclerosis, and altered blood pressure in experimental animals [64,65]. BPA may also affect heart valve functions and structure [28]. The roles of BPs in the initiation of heart pathologic states are less well known, but they have been reported to participate in the induction of various cardiac defects under experimental conditions [13]. Interestingly, despite the lack of significant statistical differences, mean concentration levels of BPA and BP-3 were clearly (about twice) higher in dogs with CDVD compared to healthy animals.

Although this fact does not justify the conclusion that there is a relationship between exposure to these compounds and the risk of developing CDVD, the present results may be a starting point for further research on the impact of BPA and BP-3 on the cardiovascular system in dogs.

In the present study, the influence of physiological factors, such as age and gender, on EDC levels was also investigated. The results showed statistically significant differences in PFOA and PFOS levels between age groups in dogs with diagnosed CDVD. It was noted that younger animals under 3 years old had the lowest mean levels of those compounds. Such results may suggest that older animals are more exposed to selected PFASs. On the other hand, such a situation may result from differences in hormonal activity and metabolism across animals of various ages, and/or from PFASs ability to accumulate in tissues and hair in older individuals, as suggested by previous studies [30,66]. However, it should be noted that the number of tested samples was very small (only three animals under 3 years old). Therefore, those observations should definitely be confirmed with studies on a vastly larger population and are insufficient to draw reliable conclusions. Moreover, no statistically significant differences in the influence of gender on the studied compounds were observed in animals with diagnosed CDVD. These results suggest no relationship between gender and exposure to the tested EDCs. This is consistent with previous studies, which also found non-statistically significant differences in gender and levels of PFAS, parabens, and BPA [30,31,32,67].

## 5. Conclusions

The present study has confirmed that dogs are exposed to various EDCs to a considerable extent, and that hair is a useful matrix for evaluating long-term exposure to these substances. Moreover, the obtained results may suggest that the degree of exposure to some EDCs (such as MeP, EtP, PrP, PFHpA and PFOA) polluting the environment may be associated with the risk of CDVD in dogs to some extent. This is evidenced by statistically significant differences in the concentrations of the above-mentioned compounds in healthy dogs and those with CDVD. Of course, further comprehensive clinical and toxicological studies are necessary to elucidate the exact relationship between dog exposure to EDCs and the risk of CDVD. These further studies should include both the identification of local factors influencing dog exposure to EDCs and investigations of pathological processes during CDVD. The present study also provides a basis for addressing environmental pollution by EDCs as a potential factor contributing to the development of diseases in companion animals.

## Figures and Tables

**Table 1:** Optimised MS/MS parameters.

Compound	Precursor Ion (*m*/*z*)	MRM 1 (Quantification) (*m*/*z*)	MRM 2 (Confirmation) (*m*/*z*)	Fragmentor (V)	Collision Energy (eV)
BPA	227	133	211.8	126	24
MeP	151	92	136	70	16
EtP	165	92	136	79	20
PrP	179	92	136	99	24
BuP	193	92	136	70	16
BP-1	215	137	105	160	16
BP-2	245	135	109	160	12
BP-3	229	151	105	160	16
BP-8	245	121	65	160	16
PFBuA	213	169.0	51.6	55	0
PFPeA	263	219.0	89.7	55	0
PFHxA	313	269.0	119.0	60	0
PFHpA	363	319.0	332.8	65	0
PFOA	413	369.0	194.3	62	0
PFOS	499	80.0	51.5	145	40
EtP-d_5_	170	92	138	80	24
BPA-d_14_	241	142	223	160	28
BP-d_10_	193	110	82	160	20
PFOA-^13^C_4_	417	371.9	172.0	77	4

MS capillary voltage 3000 V, drying-gas flow rate 9 L/min, drying-gas temperature 350 °C, and nebuliser pressure 40 psi. BPA—bisphenol A, MeP—methylparaben, EtP—ethylparaben, BuP—buthylparaben, PrP—propylparaben, BP-1—benzophenone 1, BP-2—benzophenone 2, BP-3—benzophenone 3, BP-8—benzophenone 8, PFBuA—perfluorobutanoic acid, PFPeA—perfluoropentanoic acid, PFHxA—perfluorohexanoic acid, PFHpA—perfluoroheptanoic acid, PFOA—perfluorooctanoic acid, PFOS—perfluorooctane sulfonic acid, EtP-d_5_—ethylparaben-d5, BPA-d_14_—deuterated Bisphenol A, BP-d_10_—benzophenone-d10, ^13^C_4_-PFOA—^13^C_4_-perfluorooctanoic acid.

**Table 2:** Analytical parameters of the method.

Compound	Liner Range	MDL	MQL	Recovery and Precision (RSD) (%)		Inter-Day Precision
	(ng/g)	(ng/g)	(ng/g)	Low Level	High Level	(RSD%)
BPA	4.2–700	1.25	4.2	94	95	6
MeP	2.5–1500	0.75	2.50	104	94	10
EtP	2.5–1000	0.75	2.50	99	95	7
PrP	2.0–1000	0.60	2.00	99	95	11
BuP	2.0–500	0.60	2.0	97	100	20
BP-1	2.00–625	0.50	2.0	98	102	12
BP-2	1.00–625	0.30	1.0	109	102	4
BP-3	2.00–625	0.50	2.0	92	101	4
BP-8	3.00–625	0.90	3.0	114	96	12
PFBuA	0.9–100	0.3	0.9	96	100	4
PFPeA	0.6–100	0.2	0.6	93	97	3
PFHxA	0.6–100	0.2	0.6	88	92	5
PFHpA	0.6–100	0.2	0.6	87	90	3
PFOA	0.6–100	0.2	0.6	91	95	4
PFOS	0.6–100	0.2	0.6	83	89	5

MDL: Method detection limit; MQL: Method quantification limit; RSD: Relative Standard Deviation BPA—bisphenol A, MeP—methylparaben, EtP—ethylparaben, BuP—buthylparaben, PrP—propylparaben, BP-1—benzophenone 1, BP-2—benzophenone 2, BP-3—benzophenone 3, BP-8—benzophenone 8, PFBuA—perfluorobutanoic acid, PFPeA—perfluoropentanoic acid, PFHxA—perfluorohexanoic acid, PFHpA—perfluoroheptanoic acid, PFOA—perfluorooctanoic acid, PFOS—perfluorooctane sulfonic acid.

**Table 3:** Concentration values (ng/g) and frequency of detection of EDCs in the hair samples collected from healthy dogs (H, *n* = 30) and dogs suffering from CDVD (C, *n* = 30).

	Dogs	Min	25% Per	Median	75% Per	Max	Mean (SD)	Confidence Interval 95%	Geom. Mean	% Sampl. > MDL	% Sampl > MQL	*p* Value
MeP	H *	8.16	32.48	44.20	73.78	382	76.07 (81.97)	76.1 ± 29.3 [46.7–105.4]	50.43	100	100	0.0006
	C *	24.10	63.53	105.5	207	778	162.8 (156.9)	162.8 ± 56.1 [106.7–218.9]	114	100	100	
EtP	H *	3.14	5.83	11.30	22.20	188	25.06 (41.61)	25.1 ± 14.9 [10.2–39.9]	12.88	100	100	0.0007
	C *	4.45	15.58	33.65	64.13	936	96.39 (199.6)	96.4 ± 71.4 [24.9–167.8]	35.61	100	100	
PrP	H *	<MQL	<MQL	5.38	17.13	136	15.55 (27.13)	15.6 ± 9.7 [5.8–25.3]	4.75	100	53.3	0.0001
	C *	<MQL	18.00	58.70	132	683	95.47 (132.5)	95.5 ± 47.4 [48.1–142.9]	39.68	100	90	
BuP	H	<MQL	<MQL	4.50	13.80	163	16.83 (32.36)	16.8 ± 11.6 [5.3–28.4]	5.06	100	63.3	0.0788
	C	<MQL	<MQL	13.95	47.93	560	42.64 (101.5)	42.6 ± 36.3 [6.3–78.9]	10.85	100	76.7	
BPA	H	<MQL	<MQL	<MQL	38.53	131	27.41 (39.44)	27.4 ± 14.1 [13.3–41.5]	8.13	100	43.3	0.8000
	C	<MDL	<MQL	<MQL	45.50	509	56.32 (122.7)	56.3 ± 43.9 [12.4–100.2]	8.10	93.3	40	
BP-1	H	<MDL	<MDL	<MDL	5.66	131	9.81 (25.37)	9.8 ± 7.2 [2.6–17.1]	1.16	46.6	36.6	0.5308
	C	<MDL	<MDL	<MDL	52.28	290	41.25 (76.81)	41.3 ± 4.5 [36.7–45.8]	2.17	46.6	36.6	
BP-2	H	<MDL	<MDL	<MDL	<MQL	<MQL	<MQL	-	<MDL	43.3	0	-
	C	<MDL	<MDL	<MDL	<MQL	<MQL	<MQL	-	<MDL	46.7	0	
BP-3	H	<MQL	12.00	21.75	37.93	140	33.68 (34.63)	33.7 ± 12.4 [21.3–46.1]	21.95	100	96.7	0.0616
	C	4.75	18.43	38.35	79.25	206	57.44 (54.62)	57.4 ± 19.5 [37.9–77]	35.49	100	100	
BP-8	H	<MDL	<MDL	<MDL	<MDL	6.55	<MDL	-	<MDL	13.3	10	-
	C	<MDL	<MDL	<MDL	<MDL	14.20	<MQL	-	<MDL	10	6.7	
PFBuA	H	<MQL	<MQL	1.06	1.85	12.9	2.39 (3.5)	2.4 ± 1.3 [1.1–3.6]	1.24	100	63.3	0.8950
	C	<MQL	<MQL	1.19	2.20	14.2	1.97 (2.7)	1.9 ± 1 [1–2.9]	1.19	100	63.3	
PFPeA	H	1.18	6.05	10.65	23.30	633	36.76 (113.5)	36.8 ± 40.6 [−3.9–77.4]	12.92	100	100	0.4232
	C	0.90	6.75	17.80	31.93	181	27.06 (36.80	27.1 ± 13.2 [13.9–40.2]	14.76	100	100	
PFHxA	H	<MQL	<MQL	0.97	3.41	17.90	2.91 (4.16)	2.9 ± 1.5 [1.4–4.4]	1.12	100	50	0.1713
	C	<MQL	<MQL	2.75	4.81	9.43	3.20 (2.71)	3.2 ± 1 [2.2–4.2]	1.82	100	70	
PFHpA	H *	<MQL	<MQL	<MQL	0.97	12.40	1.58 (3.17)	1.6 ± 1.1 [0.4–2.7]	0.59	100	30	0.0384
	C *	<MQL	<MQL	1.02	2.57	10.20	1.71 (2.07)	1.7 ± 0.7 [1–2.5]	0.96	100	60	
PFOA	H *	<MQL	2.16	3.13	4.60	8.65	3.62 (2.12)	3.6 ± 0.8 [2.9–4.4]	3.01	100	96.7	0.0361
	C *	0.95	2.80	4.35	6.71	9.44	4.88 (2.49)	4.9 ± 0.9 [4–5.78]	4.17	100	100	
PFOS	H	<MQL	<MQL	0.92	1.93	4.21	1.31 (1.09)	1.3 ± 0.4 [0.9–1.7]	0.93	100	73.3	0.0889
	C	<MQL	0.84	1.49	2.62	16.80	2.16 (2.94)	2.2 ± 1.1 [1.1–3.2]	1.44	100	90	

Statistically significant differences are indicated with asterisks near symbols of animal groups * with *p* < 0.05; MDL: Method detection limit; MQL: Method quantification limit; BPA—bisphenol A, MeP—methylparaben, EtP—ethylparaben, BuP—buthylparaben, PrP—propylparaben, BP-1—benzophenone 1, BP-2—benzophenone 2, BP-3—benzophenone 3, BP-8—benzophenone 8, PFBuA—perfluorobutanoic acid, PFPeA—perfluoropentanoic acid, PFHxA—perfluorohexanoic acid, PFHpA—perfluoroheptanoic acid, PFOA—perfluorooctanoic acid, PFOS—perfluorooctane sulfonic acid.

## Data Availability

The data presented in this study are available on request from the corresponding author.

## Supplementary Materials

The following supporting information can be downloaded at: https://www.mdpi.com/article/10.3390/ani15233443/s1, Table S1: Concentration values (ng/g) and frequency of detection of EDCs in the hair samples collected from healthy dogs. Y—young dogs under 3 years old (*n* = 10), M—dogs in ages between 3 and 9 y.o. (*n* = 14), O—dogs over 9 y.o. (*n* = 6); Table S2: Concentration values (ng/g) and frequency of detection of EDCs in the hair samples collected from animals with diagnosed CDVD. Y—young dogs under 3 years old (*n* = 3), M – dogs in ages between 3 and 9 y.o. (*n* = 11), O—dogs over 9 y.o. (*n* = 16); Table S3: Concentration values (ng/g) and frequency of detection of EDCs in the hair samples collected from healthy dogs. M—males (*n* = 15), F—females (*n* = 15); Table S4: Concentration values (ng/g) and frequency of detection of EDCs in the hair samples collected from animals with diagnosed CDVD. M—males (*n* = 18), F—females (*n* = 12).

## Abbreviations

BPA—bisphenol A; BP-1—benzophenone 1; BP-2—benzophenone 2; BP-3—benzophenone 3; BP-8—benzophenone 8; BPs—benzophenones; BuP—buthylparaben; CDVD—chronic degenerative valve disease; EDCs—Endocrine-disrupting chemicals; EtP—ethylparaben; MDL—method detection limit; MeP—methylparaben; MQL—method quantification limit; PBs—parabens; PFASs—per- and polyfluoroalkyl substances; PFBuA—perfluorobutanoic acid; PFHpA—perfluoroheptanoic acid; PFHxA—perfluorohexanoic acid; PFOA—perfluorooctanoic acid; PFOS—perfluorooctane sulfonic acid; PFPeA—perfluoropentanoic acid; PrP—propylparaben; RSD—relative standard deviation.
