# Supplementary material for: Could the Risk of Chronic Degenerative Valve Disease (CDVD) in Dogs Depend on Exposure to Endocrine-Disrupting Chemicals Polluting the Environment?
Source: Animals (Basel). 2025 Nov 28;15(23):3443. doi: 10.3390/ani15233443 (PMC12691118; doi:10.3390/ani15233443)
Supplement: Supplementary file 1 [file animals-15-03443-s001.zip › animals-3920756-supplementary.pdf]

Supplementary materials to:

Could the risk of chronic degenerative valve disease (CDVD) in dogs depend on exposure to endocrine-disrupting chemicals polluting the environment?

Krystyna Makowska [1], Julia Martín [2], Robert Pasławski [3], Andrzej Rychlik [1], Irene Aparicio [2], Juan Luis Santos [2], Esteban Alonso [2] Małgorzata Górecka-Politańska [4] and Sławomir Gonkowski [5]*

[1] Department of Clinical Diagnostics, Faculty of Veterinary Medicine, University of Warmia and Mazury in Olsztyn, Oczapowskiego 14, 10-957 Olsztyn, Poland
[2] Departamento de Química Analítica, Escuela Politécnica Superior, Universidad de Sevilla, C/ Virgen de África, 7, E-41011 Sevilla, Spain
[3] University of Agriculture in Krakow, Faculty of Veterinary Medicine, Mickiewicza 21, 31-120 Krakow, Poland
[4]Companion Animal Veterinary Clinic, Faculty of Veterinary Medicine, University of Warmia and Mazury in Olsztyn, Oczapowskiego 14, 10-957 Olsztyn, Poland
[5] Department of Clinical Physiology, Faculty of Veterinary Medicine, University of Warmia and Mazury in Olsztyn, Oczapowskiego 13, 10-957 Olsztyn, Poland

Table S1. Concentration values (ng/g) and frequency of detection of EDCs in the hair samples collected from healthy dogs. Y – young dogs under 3 years old (n=10), M – dogs in ages between 3 and 9 y.o. (n=14), O – dogs over 9 y.o. (n=6)

	AGE	MIN	25% PER	MEDIAN	75% PER	MAX	MEAN (SD)	CONFIDENCE INTERVAL 95%	GEOM. MEAN	% SAMPL >MDL	% SAMPL >MQL	P VALUE
MeP	Y	12.2	26.9	42.55	64.05	284	73.76 (85.65)	73.7 ± 53.1 [20.7-126.8]	45.73	100	100	.073
	M	8.16	43.85	65.75	109.33	382	97.1 (95.22)	97.1 ± 49.9 [47.2- 147]	67.88	100	100	
	O	15.1	21.98	31.65	34.43	72.6	34.18 (20.4)	34.2 ± 16.3 [17.9- 50.5]	30.04	100	100	
EtP	Y	3.14	4.42	9.51	24.98	151	29.44 (46.23)	29.4 ± 28.6 [0.8- 58.1]	12.53	100	100	.450
	M	3.56	10.5	14.6	19.29	188	27.04 (46.99)	27 ± 24.6 [2.4- 51.6]	15.35	100	100	
	O	4.21	5	7.2	11.06	43.7	13.15 (15.23)	13.2 ± 12.2 [1- 25.3]	8.96	100	100	
PrP	Y	<MQL	<MQL	4.91	12.83	34.9	10.43 (12.73)	10.4 ± 7.9 [2.5- 18.3]	4.1	100	16.67	.107
	M	<MQL	<MQL	11.08	32.35	136	24.32 (36.67)	24.3 ± 19.2 [5.1- 43.5]	8.36	100	71.43	
	O	<MQL	<MQL	<MQL	<MQL	16.8	3.63 (6.45)	3.6 ± 5.2 [-1.5- 8.8]	1.6	100	50	
BuP	Y*	<MQL	<MQL	6.66	46.68	163	32.99 (51.35)	33 ± 31.8 [1.2- 64.8]	7.43	100	33.33	.039
	M*	<MQL	2.99	7.87	12.2	47.3	11.58 (13.39)	11.6 ± 7 [4.6- 18.6]	6.22	100	78.57	
	O*	<MDL	<MQL	<MQL	<MQL	4.7	1.52 (1.57)	1.5 ± 1.3 [0.3- 2.8]	1.12	100	60	
BPA	Y	<MQL	<MQL	<MQL	31.6	76.3	20.11 (28.47)	20.1 ± 17.6 [2.5- 37.8]	6.69	100	50	.990

	M	<MQL	<MQL	<MQL	38	131	33.26 (48.39)	33.3 ± 25.3 [7.9- 58.6]	8.82	100	42.86	
	O	<MQL	<MQL	13.9	28.48	94	25.9 (35.63)	25.9 ± 28.5 [-2.6- 54.4]	9.33	100	40	
BP-1	Y	<MDL	<MQL	<MQL	2.67	12.2	2.24 (3.73)	2.2 ± 2.3 [-0.1- 4.5]	<MQL	33.3	33.33	.796
	M	<MDL	<MDL	<MQL	12.96	131	16.51 (35.77)	16.5 ± 18.7 [-2.2- 35.2]	<MQL	50	57.14	
	O	<MDL	<MDL	<MDL	8.69	28.3	6.8 (11.45)	6.8 ± 9.2 [-2.4- 16]	<MQL	50	30	
BP-2	Y	<MDL	<MDL	<MQL	<MQL	<MQL	<MQL	-	<MQL	16.67	0	.250
	M	<MDL	<MDL	<MDL	<MQL	<MQL	<MQL	-	<MDL	42.86	0	
	O	<MDL	<MDL	<MDL	<MDL	<MQL	<MDL	-	<MDL	0	0	
BP-3	Y	<MQL	12.3	21.75	29.8	134	32.15 (38.07)	32.2 ± 23.6 [8.6- 55.7]	18.49	100	100	.797
	M	8.45	10.78	22.15	33.4	140	31.46 (34.48)	31.5 ± 18.1 [13.4- 49.5]	22.16	100	100	
	O	9.27	13.15	36.65	61.43	90.7	41.38 (34.12)	41.4 ± 27.3 [14.1- 68.7]	28.57	100	90	
BP-8	Y	<MDL	<MDL	<MDL	<MDL	<MQL	<MQL	-	<MDL	33.33	16.67	.194
	M	<MDL	<MDL	<MDL	<MDL	6.55	<MQL	-	<MDL	14.29	14.29	
	O	<MDL	<MDL	<MDL	1.24	4.77	<MQL	-	<MDL	0	0	
PFBuA	Y	<MQL	1	1.24	1.55	12.4	2.3 (3.59)	2.3 ± 2.2 [0.1- 4.5]	1.34	100	50	.726
	M	<MQL	<MQL	1.07	2.3	12.9	2.47 (3.52)	2.5 ± 1.8 [0.6- 4.3]	1.24	100	57.14	
	O	<MQL	<MQL	<MQL	1.54	10.2	2.37 (3.87)	2.4 ± 3.1 [-0.7- 5.5]	1.07	100	80	
PFPeA	Y	8.88	8.415	16.35	27.23	633	78.23 (195.26)	78.2 ± 121 [-42.8- 199.2]	19.06	100	100	.597
	M	4.16	5.79	8.96	16	48.5	15.2 (14.37)	15.2 ± 2.3 [12.9- 17.5]	10.87	100	100	
	O	1.18	6.16	12.88	20.18	54.3	17.96 (19.31)	18 ± 15.5 [2.5- 33.4]	10.10	100	100	
PFHxA	Y	<MQL	<MQL	1.87	4.79	8.96	2.93 (3.14)	2.9 ± 1.9 [1- 4.9]	1.4	100	16.67	.187
	M	<MQL	<MQL	1.78	3.75	17.9	3.83 (5.32)	3.8 ± 2.8 [1- 6.6]	1.44	100	57.14	
	O	<MQL	<MQL	<MQL	<MQL	2.89	.73 (1.06)	0.7 ± 0.8 [-0.1- 1.6]	<MQL	100	60	
PFHpA	Y	<MQL	<MQL	<MQL	1.24	12.1	1.87 (3.67)	1.9 ± 2.3 [-0.4- 4.1]	.69	100	0	.222
	M	<MQL	<MQL	<MQL	1.73	12.4	1.91 (3.48)	1.9 ± 1.8 [0.1- 3.7]	.69	100	35.71	
	O	<MQL	<MQL	<MQL	<MQL	<MQL	<MQL	-	<MQL	100	40	
PFOA	Y	1.24	1.59	2.6	4.93	8.65	3.72 (2.82)	3.7 ± 1.7 [2- 5.5]	2.92	100	83.33	.318
	M	1.97	2.49	4.05	4.79	8.21	4.03 (1.78)	4 ± 0.9 [3.1- 5]	3.68	100	100	
	O	<MQL	2.27	2.69	3.27	3.71	2.5 (1.21)	2.5 ± 1 [1.5- 3.5]	1.98	100	100	
PFOS	Y	<MQL	<MQL	<MQL	1.55	4.04	1.11 (1.21)	1.1 ± 0.7 [0.4- 1.9]	.7	100	66.67	.129
	M	<MQL	.79	1.32	2.23	4.21	1.67 (1.13)	1.7 ± 0.6 [1.1- 2.3]	1.33	100	92.86	
	O	<MQL	<MQL	.82	.97	1.5	.79 (.46)	0.8 ± 0.4	.67	100	50	

[0.4- 1.2]

Statistically significant differences are indicated with asterisks near symbols of animal groups * with p<0.05; MDL: Method detection limit; MQL: Method quantification limit; BPA – bisphenol A, MeP – methylparaben, EtP – ethylparaben, BuP – buthylparaben, PrP – propylparaben, BP-1 - benzophenone 1, BP-2 - benzophenone 2, BP-3 - benzophenone 3, BP-8 - benzophenone 8, PFBuA - perfluorobutanoic acid, PFPeA - perfluoropentanoic acid, PFHxA - perfluorohexanoic acid, PFHpA - perfluoroheptanoic acid, PFOA - perfluorooctanoic acid, PFOS - perfluorooctane sulfonic acid

Table S2. Concentration values (ng/g) and frequency of detection of EDCs in the hair samples collected from animals with diagnosed CDVD. Y – young dogs under 3 years old (n=3), M – dogs in ages between 3 and 9 y.o. (n=11), O – dogs over 9 y.o. (n=16)

	AGE	MIN	25% PER	MEDIAN	75% PER	MAX	MEAN (SD)	CONFIDENCE INTERVAL 95%	GEOM. MEAN	% SAMPL >MDL	% SAMPL >MQL	P VALUE
MeP	Y	24.1	102.5	180	301	422	208.7 (200.5)	208.7 ± 226.9 [-18.2- 435.6]	122.33	100	100	.947
	M	26.1	47.75	88.8	280.5	355	151.17 (128.31)	151.2 ± 75.8 [75.3- 227]	102.95	100	100	
	O	46.7	67.95	105.5	186.75	778	162.13 (175.28)	162.1 ± 85.9 [76.2- 248]	120.6	100	100	
EtP	Y	39.5	44.25	49	72.75	96.5	61.67 (30.54)	61.7 ± 34.6 [27.1- 96.2]	57.16	100	100	.353
	M	4.45	14.3	21	48.95	596	85.9 (173.51)	85.9 ± 102.5 [-16.6- 188.4]	29.68	100	100	
	O	4.67	15.58	34.05	62.25	936	110.12 (237.26)	110.1 ± 116.3 [-6.1- 226.4]	36.94	100	100	
PrP	Y	68.5	81.9	95.3	135.15	175	112.93 (55.4)	112.9 ± 62.7 [50.2- 175.6]	104.54	100	100	.205
	M	<MQL	6.21	18.4	109.35	210	62.34 (73.06)	62.3 ± 43.2 [19.2- 105.5]	19.78	100	81.82	
	O	<MQL	33.03	58.7	116.25	683	114.98 (169.35)	115 ± 83 [32- 198]	53.39	100	93.75	
BuP	Y	<MQL	7.7	14.4	34.35	54.3	23.23 (27.73)	23.2 ± 31.4 [-8.1- 54.6]	9.21	100	66.67	.742
	M	<MQL	<MQL	4.06	46.7	765.7	23.31 (30.59)	23.3 ± 18.1 [5.2- 41.4]	6.84	100	72.73	
	O	<MQL	6.55	20.65	44.8	560	59.58 (136.16)	59.6 ± 66.7 [-7.1- 126.3]	15.36	100	81.25	
BPA	Y	<MQL	24.25	46.4	74.7	103	50.5 (50.57)	50.5 ± 57.2 [-6.7- 107.7]	21.57	100	66.67	.158
	M	<MDL	<MQL	<MQL	<MQL	75.7	11.09 (22.91)	11.1 ± 13.5 [-2.4- 24.6]	3.31	90.91	18.19	
	O	<MDL	<MQL	8.65	69.4	509	88.48 (160.59)	88.5 ± 78.7 [9.8- 167.2]	12.47	93.75	50	
BP-1	Y	42	83.5	125	148	171	112.67 (65.38)	112.7 ± 74 [38.7- 186.6]	96.47	100	100	.071
	M	<MDL	<MDL	<MDL	9.5	239	32.02 (73.88)	3.6 ± 4.8 [-1.2- 8.4]	1.51	45.45	27.27	
	O	<MDL	<MDL	<MDL	10.81	290	34.29 (77.87)	34.3 ± 38.2 [-3.9- 72.4]	1.36	37.5	31.25	
BP-2	Y	<MQL	<MQL	<MQL	<MQL	<MQL	<MQL	-	<MQL	100	0	.083
	M	<MDL	<MDL	<MDL	<MQL	<MQL	<MQL	-	<MQL	27.27	0	
	O	<MDL	<MDL	<MQL	.5	<MQL	<MQL	-	<MQL	50	0	

BP-3	Y	30.5	42.15	53.8	69.15	84.5	56.27 (27.08)	56.3 ± 30.6 [25.6- 86.9]	51.76	100	100	.633
	M	7.12	29.8	38.5	73	157	55.97 (43.85)	56 ± 26.5 [29.5- 82.5]	40.3	100	100	
	O	4.75	12.11	33.75	70.45	206	58.67 (66.22)	58.7 ± 32.4 [26.2- 91.1]	30.3	100	100	
BP-8	Y	<MDL	<MDL	<MDL	<MDL	<MQL	<MQL	-	<MQL	0	0	.245
	M	<MDL	<MDL	<MDL	<MDL	<MQL	<MQL	-	<MQL	0	0	
	O	<MDL	<MDL	<MDL	<MDL	14.2	1.59 (3.48)	1.6 ± 1.7 [-0.1- 3.3]	<MQL	18.75	12.5	
PFBuA	Y	<MQL	<MQL	1.12	1.19	1.26	.94 (.43)	0.9 ± 0.5 [0.4- 1.4]	.86	100	66.67	.441
	M	<MQL	<MQL	<MQL	1.81	14.2	2.1 (4.05)	2.1 ± 2.4 [-0.3- 4.5]	1.01	100	45.45	
	O	<MQL	<MQL	1.33	3.13	5.08	<MQL	-	1.43	100	75	
PFPeA	Y	6.79	11.34	15.9	20.45	25	11.12 (12.3)	11.1 ± 13.9 [-2.8- 25]	13.03	100	100	.461
	M	3.97	7.24	17.6	29.25	181	31.25 (50.77)	31.3 ± 30 [1.2- 61.2]	15.92	100	100	
	O	.9	8.59	18.95	35.55	116	27.16 (28.56)	27.2 ± 14 [13.2- 41.1]	16.37	100	100	
PFHxA	Y	<MQL	<MQL	<MQL	3.67	7.23	2.61 (4.00)	2.6 ± 4.5 [-1.9- 7.1]	.87	100	33.33	.654
	M	<MQL	2.22	3.1	5.44	7.36	3.53 (2.4)	3.5 ± 1.4 [2.1- 4.9]	2.42	100	81.82	
	O	<MQL	<MQL	2.6	4.19	9.43	3.09 (2.83)	3.1 ± 1.4 [1.7- 4.5]	1.73	100	68.75	
PFHpA	Y	<MQL	1.48	2.65	2.7	2.75	1.9 (1.39)	1.9 ± 1.6 [0.3- 3.5]	1.3	100	66.67	.647
	M	<MQL	.685	1.28	2.19	2.55	1.37 (.83)	1.4 ± 0.5 [0.9- 1.9]	1.09	100	81.82	
	O	<MQL	<MQL	<MQL	2.75	10.2	1.91 (2.73)	1.9 ± 1.3 [0.6- 3.2]	.83	100	43.75	
PFOA	Y*	1.63	1.74	1.84	2.65	3.45	2.31 (1.00)	2.3 ± 1.1 [1.2- 3.4]	2.18	100	100	.007
	M*	1.12	6.12	6.93	8.37	9.44	6.6 (2.57)	6.6 ± 1.5 [5.1- 8.1]	5.81	100	100	
	O*	.954	2.8	3.91	5.58	8.39	4.18 (1.85)	4.2 ± 0.9 [3.3- 5.1]	3.75	100	100	
PFOS	Y*	<MQL	<MQL	.68	.68	.68	<MQL	-	<MQL	100	86.67	.028
	M*	<MQL	<MQL	1.81	1.74	16.8	3.33 (4.6)	3.3 ± 2.7 [0.6- 6]	2	100	90.91	
	O*	<MQL	1.31	1.42	2.97	3.59	1.65 (.92)	1.7 ± 0.4 [1.2- 2.1]	1.4	100	93.75	

Statistically significant differences are indicated with asterisks near symbols of animal groups * with $p<0.05$; MDL: Method detection limit; MQL: Method quantification limit; BPA – bisphenol A, MeP – methylparaben, EtP – ethylparaben, BuP – buthylparaben, PrP – propylparaben, BP-1 - benzophenone 1, BP-2 - benzophenone 2, BP-3 - benzophenone 3, BP-8 - benzophenone 8, PFBuA - perfluorobutanoic acid, PFPeA - perfluoropentanoic acid, PFHxA - perfluorohexanoic acid, PFHpA - perfluoroheptanoic acid, PFOA - perfluorooctanoic acid, PFOS - perfluorooctane sulfonic acid

Table S3. Concentration values (ng/g) and frequency of detection of EDCs in the hair samples collected from healthy dogs. M – males (n=15), F – females (n=15)

	SEX	MIN	25% PER	MEDIAN	75% PER	MAX	MEAN (SD)	CONFIDENCE INTERVAL 95%	GEOM. MEAN	% SAMPL >MDL	% SAMPL >MQL	P VALUE
MeP	M	8.16	34.45	43.5	74.95	284	74.82 (74.94)	74.8 ± 38 [36.9- 112.7]	50.99	100	100	.868
	F	12.2	27.25	60.8	71.1	382	78.66 (94.11)	78.7 ± 47.6 [31- 126.3]	50.11	100	100	
EtP	M	3.56	6.59	12	34.8	188	37.49 (56.37)	37.5 ± 28.5 [9- 66]	16.54	100	100	.407
	F	3.14	5.75	10.6	16.1	35.4	12.63 (8.84)	12.6 ± 4.5 [8.2- 17.1]	10.03	100	100	
PrP	M	<MQL	<MQL	2.94	13.6	34.9	9.37 (11.11)	9.4 ± 5.6 [3.7- 15]	4.0	100	53.3	.615
	F	<MQL	<MQL	7.81	27.6	136	21.73 (36.32)	21.7 ± 18.4 [3.3- 40.1]	5.64	100	53.3	
BuP	M	<MQL	<MQL	3.55	40.4	163	26.18 (43.70)	26.12 ± 22.1 [4.1- 48.3]	5.81	100	66.67	.64
	F	<MQL	<MQL	5.75	10.34	34.3	7.23 (8.67)	7.2 ± 4.4 [2.8- 11.6]	3.78	100	60.0	
BPA	M	<MQL	<MQL	25.7	69.35	130	37.96 (42.57)	38 ± 21.5 [16.4- 59.5]	13.89	100	60.0	.09
	F	<MQL	<MQL	2.1	11.65	131	16.85 (34.23)	16.9 ± 17.3 [-0.5- 34.2]	4.76	100	26.67	
BP-1	M*	<MDL	<MQL	<MQL	14	131	16.04 (34.06)	16 ± 17.2 [-1.2- 33.3]	2.58	73.3	46.67	.012
	F*	<MDL	<MDL	<MDL	<MDL	36.1	3.58 (9.51)	3.6 ± 4.8 [-1.2- 8.4]	0.52	20.0	20.0	
BP-2	M	<MDL	<MDL	<MQL	<MQL	<MQL	<MQL	-	<MQL	53.33	0	-
	F	<MDL	<MDL	<MDL	<MQL	<MQL	<MQL	-	<MQL	33.33	0	
BP-3	M*	8.45	17.3	30.7	60.95	140	47.59 (42.92)	47.6 ± 21.7 [25.9- 69.3]	33.06	100	100	.040
	F*	<MQL	10.1	13.3	26.95	62.3	19.76 (15.06)	19.8 ± 7.6 [12.1- 27.4]	14.57	100	93.33	
BP-8	M	<MDL	<MDL	<MDL	<MDL	<MQL	<MQL	-	<MQL	13.33	0	-
	F	<MDL	<MDL	<MDL	<MDL	6.55	<MQL	-	<MQL	13.33	13.33	
PFBuA	M	<MQL	<MQL	1	1.86	12.4	2.67 (3.82)	2.7 ± 1.9 [0.7- 4.6]	1.28	100	66.67	1
	F	<MQL	<MQL	1.11	1.62	12.9	2.11 (3.22)	2.1 ± 1.6 [0.5- 3.7]	1.19	100	60.0	
PFPeA	M	5.44	8.415	17.5	35.4	633	61.31 (158.95)	61.3 ± 9.6 [51.7- 70.9]	19.64	100	100	.042
	F	1.18	5.43	7.71	16.35	38.4	12.21 (11.06)	12.2 ± 5.6 [6.6- 17.8]	8.49	100	100	
PFHxA	M	<MQL	<MQL	1.78	3.1	12.1	2.83 (3.51)	2.8 ± 1.8 [1- 4.6]	1.31	100	60	.626
	F	<MQL	<MQL	<MQL	3.58	17.9	2.99 (4.86)	3 ± 2.5 [0.5- 5.4]	.96	100	40	
PFHpA	M	<MQL	<MQL	<MQL	<MQL	12.1	1.69 (3.34)	1.7 ± 1.7 [-0.0- 3.4]	.58	100	26.67	.798
	F	<MQL	<MQL	<MQL	1.11	12.4	1.46 (3.1)	1.5 ± 1.6 [-0.1- 3]	.59	100	40	
PFOA	M	1.24	2.43	3.43	5.19	8.65	4.04 (2.24)	4 ± 1.1 [2.9- 5.2]	3.52	100	100	.3
	F	<MQL	2.06	2.67	4.41	8.39	3.19 (1.97)	3.2 ± 1 [2.2- 4.2]	2.57	100	93.3	

	SEX	MIN	25% PER	MEDIAN	75% PER	MAX.	MEAN (SD)	CONFIDENCE INTERVAL 95%	GEOM. MEAN	% SAMPL >MDL	% SAMPL >MQL	P VALUE
PFOS	M	<MQL	.72	.99	1.86	4.21	1.33 (1.1)	1.3 ± 0.6 [0.8- 1.9]	.99	100	80	.69
	F	<MQL	<MQL	.89	1.74	4.04	1.28 (1.13)	1.3 ± 0.6 [0.7- 1.8]	.88	100	66.67	

Statistically significant differences are indicated with asterisks near symbols of animal groups * with $p<0.05$; MDL: Method detection limit; MQL: Method quantification limit; BPA – bisphenol A, MeP – methylparaben, EtP – ethylparaben, BuP – buthylparaben, PrP – propylparaben, BP-1 - benzophenone 1, BP-2 - benzophenone 2, BP-3 - benzophenone 3, BP-8 - benzophenone 8, PFBuA - perfluorobutanoic acid, PFPeA - perfluoropentanoic acid, PFHxA - perfluorohexanoic acid, PFHpA - perfluoroheptanoic acid, PFOA - perfluorooctanoic acid, PFOS - perfluorooctane sulfonic acid

Table S4. Concentration values (ng/g) and frequency of detection of EDCs in the hair samples collected from animals with diagnosed CDVD. M – males (n=18), F –females (n=12)

	SEX	MIN	25% PER	MEDIAN	75% PER	MAX.	MEAN (SD)	CONFIDENCE INTERVAL 95%	GEOM. MEAN	% SAMPL >MDL	% SAMPL >MQL	P VALUE
MeP	M	26.1	72.65	125.5	241	422	163.76 (119.9)	163.8 ± 60.7 [103.1- 224.4]	122.97	100	100	.409
	F	24.1	57	83.45	183	778	161.29 (206.55)	161.3 ± 104.5 [56.8- 265.8]	101.68	100	100	
EtP	M	4.67	18.33	34.7	65.18	596	91.2 (151.97)	91.2 ± 76.9 [14.3- 168.1]	40.84	100	100	.363
	F	4.45	14.93	19.65	39.88	936	104.18 (263.09)	104.2 ± 133.1 [-29- 237.3]	29	100	100	
PrP	M	<MQL	22.375	58.7	142	683	108.13 (157.27)	108.1 ± 79.6 [28.5- 187.7]	40.94	100	88.89	.783
	F	<MQL	18.45	58.5	92.6	306	76.48 (85.86)	76.5 ± 43.5 [33- 119.9]	37.85	100	91.67	
BuP	M	<MQL	22.38	15.05	55.45	560	57.87 (129.13)	57.9 ± 65.3 [-7.5- 123.2]	12.87	100	72.22	.482
	F	<MQL	2.64	8.48	37.35	54.3	19.8 (21.08)	19.8 ± 10.7 [9.1- 30.5]	8.4	100	83.33	
BPA	M	<MDL	<MQL	2.1	11.93	202	23.34 (51.32)	23.3 ± 26 [-2.6- 49.3]	4.81	94.44	27.78	.118
	F	<MDL	<MQL	26.5	92.28	509	105.76 (176.51)	105.8 ± 89.3 [16.4- 195.1]	17.68	91.67	58-33	
BP-1	M	<MDL	<MDL	<MDL	<MQL	239	31.38 (71.18)	31.4 ± 36 [-4.6- 67.4]	1.2	38.89	27.78	.27
	F	<MDL	<MDL	20.45	85.25	290	56.16 (85.57)	56.2 ± 43.3 [12.9- 99.5]	5.26	91.67	50.0	
BP-2	M	<MDL	<MDL	<MDL	<MQL	<MQL	<MDL	-	<MDL	38.89	0	-
	F	<MDL	<MDL	<MQL	<MQL	<MQL	<MQL	-	<MQL	58.33	0	
BP-3	M	4.75	8.92	41.4	93.7	206	64.54 (63.23)	64.5 ± 32 [32.5- 96.5]	34.52	100	100	.882
	F	10.1	24.5	36.9	46.68	153	46.79 (38.47)	46. ± 19.5 [27.3- 66.3]	36.99	100	100	
BP-8	M	<MDL	<MDL	<MDL	<MDL	3.95	<MDL	-	<MDL	11.11	5.56	-
	F	<MDL	<MDL	<MDL	<MDL	14.2	<MQL	-	<MDL	8.33	8.33	
PFBuA	M	<MQL	<MQL	1.32	2.86	14.2	2.4 (3.26)	2.4 ± 1.6 [0.7- 4]	1.38	100	66.67	.351
	F	<MQL	<MQL	.95	1.54	4.99	1.34 (1.31)	1.3 ± 0.7 [0.7- 2]	.96	100	83.33	

PFPeA	M	5.92	8.8	21.45	34.4	181	36.07 (44.77)	36.1 ± 22.7 [13.4- 58.7]	21.83	100	100	.197
	F	.9	4.46	10.7	22.45	36.6	13.54 (11.84)	13.5 ± 6 [7.5- 19.5]	9.53	100	100	
PFHxA	M	<MQL	2.03	3.14	5.75	9.43	3.79 (2.95)	3.8 ± 1.5 [2.3- 5.3]	2.32	100	77.78	.213
	F	<MQL	<MQL	2.47	3.42	6.59	2.32 (2.1)	2.3 ± 1.1 [1.3- 3.4]	1.27	100	83.33	
PFHpA	M	<MQL	<MQL	1.02	2.475	10.2	1.91 (2.45)	1.9 ± 1.2 [0.7- 3.1]	1.05	100	66.67	.71
	F	<MQL	<MQL	.79	2.4	4.27	1.41 (1.37)	1.4 ± 0.7 [0.7- 2.1]	.83	100	50	
PFOA	M	.954	3.35	5.08	7.13	9.44	5.21 (2.64)	5.2 ± 1.3 [3.9- 6.6]	4.41	100	100	.385
	F	1.63	2.62	3.74	6.25	8.39	4.39 (2.27)	4.4 ± 1.1 [3.2- 5.5]	3.84	100	100	
PFOS	M	<MQL	.97	1.59	2.61	16.8	2.54 (3.66)	2.5 ± 1.8 [0.7- 4.4]	1.65	100	94.44	.433
	F	<MQL	.7	1.23	2.06	3.94	1.59 (1.23)	1.6 ± 0.6 [1- 2.2]	1.17	100	83.33	

Statistically significant differences are indicated with asterisks near symbols of animal groups * with $p<0.05$; MDL: Method detection limit; MQL: Method quantification limit; BPA – bisphenol A, MeP – methylparaben, EtP – ethylparaben, BuP – buthylparaben, PrP – propylparaben, BP-1 - benzophenone 1, BP-2 - benzophenone 2, BP-3 - benzophenone 3, BP-8 - benzophenone 8, PFBuA - perfluorobutanoic acid, PFPeA - perfluoropentanoic acid, PFHxA - perfluorohexanoic acid, PFHpA - perfluoroheptanoic acid, PFOA - perfluorooctanoic acid, PFOS - perfluorooctane sulfonic acid
